# Live Monitoring of Strain‐Promoted Azide Alkyne Cycloadditions in Complex Reaction Environments by Inline ATR‐IR Spectroscopy

**DOI:** 10.1002/chem.201905478

**Published:** 2020-03-13

**Authors:** Dennis Svatunek, Gottfried Eilenberger, Christoph Denk, Daniel Lumpi, Christian Hametner, Günter Allmaier, Hannes Mikula

**Affiliations:** ^1^ Institute of Applied Synthetic Chemistry TU Wien Getreidemarkt 9 1060 Vienna Austria; ^2^ Institute of Chemical Technologies and Analytics TU Wien Getreidemarkt 9 1060 Vienna Austria

**Keywords:** bioorthogonal chemistry, click chemistry, IR spectroscopy, kinetics, reaction monitoring

## Abstract

The strain‐promoted azide alkyne cycloaddition (SPAAC) is a powerful tool for forming covalent bonds between molecules even under physiological conditions, and therefore found broad application in fields ranging from biological chemistry and biomedical research to materials sciences. For many applications, knowledge about reaction kinetics of these ligations is of utmost importance. Kinetics are commonly assessed and studied by NMR measurements. However, these experiments are limited in terms of temperature and restricted to deuterated solvents. By using an inline ATR‐IR probe we show that the cycloaddition of azides and alkynes can be monitored in aqueous and even complex biological fluids enabling the investigation of reaction kinetics in various solvents and even human blood plasma under controlled conditions in low reaction volumes.

## Graphical abstract

**Taking a closer look**: We show that azide cycloadditions can be monitored in complex reaction environments by using an inline ATR‐IR probe in a small‐volume reaction flask also facilitating full temperature control. This method enables following bioorthogonal azide cyclooctyne ligations even in biological fluids such as human plasma.

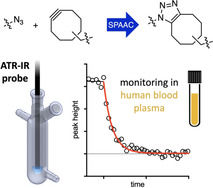

The 1,3‐dipolar cycloaddition of organic azides and alkynes, first reported by Huisgen in 1960,^1^ has been re‐emerging since the development of copper‐catalyzed click chemistry by Sharpless^2^ and Meldal,^3^ which has found broad application in many fields and become a robust and efficient tool for bioconjugation.^4^, ^5^, ^6^ However, due to the need for cytotoxic copper, these reactions are only of limited suitability for in vivo applications.^7^ Also decades ago, in 1961 Wittig and co‐workers already reported that cyclooctyne and phenyl azide react extremely fast at room temperature forming a single product.^8^ Based on these findings Bertozzi and co‐workers developed the concept of copper‐free and thus bioorthogonal click chemistry.^9^ Due to the strained triple bond, cyclooctynes are already distorted towards transition state geometry, which significantly lowers the activation energy.^10^, ^11^ Strain‐promoted azide alkyne cycloaddition (SPAAC) reactions thus proceed already at room temperature without the use of any catalyst.^12^ Several cyclooctyne derivatives have been prepared in the last decade to improve both reactivity and stability of these bioorthogonal compounds.^13^, ^14^, ^15^, ^16^ In addition, the influence of different azides was investigated.^9^, ^13^, ^14^, ^15^, ^17^, ^18^, ^19^, ^20^, ^21^ Reported second order rate constants range from 2.4×10^−3^ m ^−1^ s^−1^ up to 34 m ^−1^ s^−1^.^9^, ^22^ Knowledge about the kinetics of bioorthogonal ligations in complex environments such as biological fluids is of utmost importance considering respective applications in vitro and in vivo. However, commonly used methods do not offer a general approach for the measurement in biological fluids of any SPAAC reaction.

Reaction kinetics are commonly assessed and investigated by NMR measurements.^9^, ^13^, ^16^, ^21^, ^23^, ^24^, ^25^, ^26^ In this case the reaction partners are mixed in deuterated solvents in an NMR tube and the reaction is monitored by consecutive scans at defined time points. The advantage of this method is the ability to easily follow every involved species, assuming separated signals. However, there are several drawbacks. Control of the reaction temperature is difficult and limited, and the need for deuterated solvents renders measurements in complex biological fluids impossible. In addition to NMR, UV–Vis has been successfully used to study SPAAC reaction kinetics.^27^, ^28^, ^29^ While this approach can be used for live reaction monitoring with the possibility of temperature control and the use of a wide variety of solvents, structural requirements are imposed on the reaction partners, and solvents with highly interfering background, such as biological fluids, cannot be used. Furthermore, fluorescence measurements have been applied for the investigation of click kinetics.^30^ However, these methods depend on fluorogenic reactants or fluorescence quenching during the formation of the ligation product, and can thus not be used as general analytical tools. Very recently, Steflova et al. have reported the stepwise investigation of SPAAC by capillary electrophoresis.^31^

Herein we present a method for the live monitoring of SPAAC ligations at different temperatures and in various solvents, including human blood plasma, using inline ATR‐IR spectroscopy (Figure 1). IR spectroscopy offers a monitoring of the reaction progress by following the characteristic absorption of the azide moiety at around 2100 cm^−1^, which is usually well separated from signals of other functional groups and solvents. Figure 2 a shows the IR spectra of phenylacetylene (**1**), phenyl azide (**2**) and the respective click product **3**, with a separated azide double band^32^ around 2100 cm^−1^. Determination of reaction rates of azide cycloadditions using IR spectroscopy was already performed by Huisgen et al. in 1967.^33^ They were able to determine the reactivity of over 40 different alkynes and alkenes in the 1,3‐dipolar cycloaddition with azides. More recently, van Delft and co‐workers used transmission‐FTIR measurements to investigate the significantly higher reactivity of electron‐deficient aryl azides towards aliphatic cyclooctynes.^19^ However, although they have been successful to study reactions carried out in a 9:1 mixture of THF and H_2_O, measurements at higher water content or in other solvents like methanol failed.

**Figure 1:**
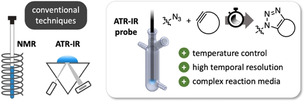
In contrast to conventional methods inline ATR‐IR enables monitoring of SPAAC under controlled reaction conditions and in complex environments.

**Figure 2:**
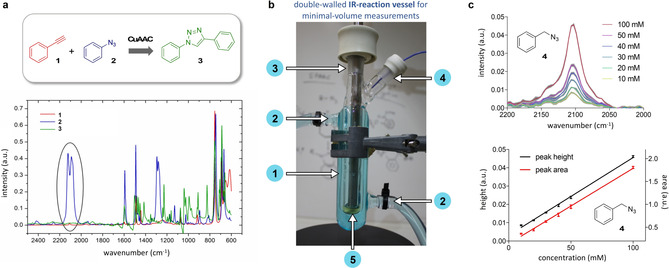
a) Comparison of IR spectra of phenylacetylene (**1**), phenyl azide (**2**) and the respective click product **3**, highlighting the separated azide double band^31^ of **2**. b) Reaction flask for inline ATR‐IR measurements (1: double walled tube, 2: thermostat, 3: ATR‐IR probe is inserted through the NS14 glass joint on top, 4: temperature sensor (blue cable) and/or addition of reagents, 5: magnetic stirring of reaction volumes as low as 0.5 mL). c) Inline ATR‐IR measurements showing the azide band of **2** at different concentrations (DMSO, 37 °C, gray areas indicate SD, *n*=3), and the resulting correlations of peak height and area to the concentration of **2**.

To address the limitations of currently used methods we aimed to design a new system and strategy enabling the monitoring of azide cycloadditions in aqueous and more complex solutions with full control of the reaction temperature. To this end, a ReactIR 15 system (Mettler Toledo) equipped with an ATR‐IR SiComp probe was used, which features a silicon crystal for ATR that (in contrast to diamond crystals) exhibits only low absorption around 2100 cm^−1^. This setup not only allows for temperature control, but furthermore the use of an inert gas atmosphere and stirring, by simply immerging the probe into a reaction solution within a tempered and sealed vessel. For this study, we have used a special flask (Figure 2 b) to enable temperature‐controlled measurements in low reaction volumes. It consists of a double‐walled tube connected to a thermostat, and two NS14 glass joints arranged in a 45° angle, one on top for inserting the ATR‐IR probe and a second one for a temperature sensor and/or the addition of reagents. With this setup a volume of 0.5 mL is sufficient for reaction monitoring while stirring. In case stirring is not required even lower volumes can be used.

The first evaluation of the setup was done by measuring benzyl azide (**2**) in acetonitrile at concentrations ranging from 10 to 100 mm providing excellent linear correlation between the peak height and area of the azide band to the concentration of **2** (Figure 2 c).

We next applied this setup to the monitoring of a SPAAC in a reaction volume of 1 mL. Before starting the measurement, the background of pure solvent was acquired. The azide solution (0.9 mL) was then placed in the flask and upon temperature equilibration a 10‐fold concentrated solution of the cyclooctyne (0.1 mL) was added to obtain an equimolar mixture of both reagents. Monitoring of the reaction was started before adding the second reactant and reaction was followed by consecutive inline ATR‐IR spectroscopy. The interval between the scans was chosen based on the reaction rate, ranging from 15 s for fast reactions to 1 min for slower conversions. For detailed description of the used settings see Supporting Information. For evaluation of this setup the reaction between benzyl azide (**4**) and cyclooctyne (**5**) in DMSO at 37 °C was monitored (Figure 3 a). Data was recorded and pre‐processed using the iC IR™ 4.3.27 software (Mettler Toledo) and analyzed in Prism 6 (GraphPad Software Inc.). First, the background spectrum was subtracted followed by baseline correction. Then a peak region was assigned to the area of the azide signal (2100 cm^−1^). Peak area and peak height were analyzed over time and rate constants were determined by linearization and subsequent linear fitting (Figure 3 b). Reaction monitoring using the signal height showed less noise and a better correlation. In addition, the peak height is not dependent on the width of the assigned peak region.^30^ Therefore, peak height was chosen for further measurements. Despite the different signal‐to‐noise ratios similar results were obtained for the calculated rate constants (peak height: 1.52×10^−2^ m ^−1^ s^−1^ vs. peak area: 1.46×10^−2^ m ^−1^ s^−1^).

**Figure 3:**
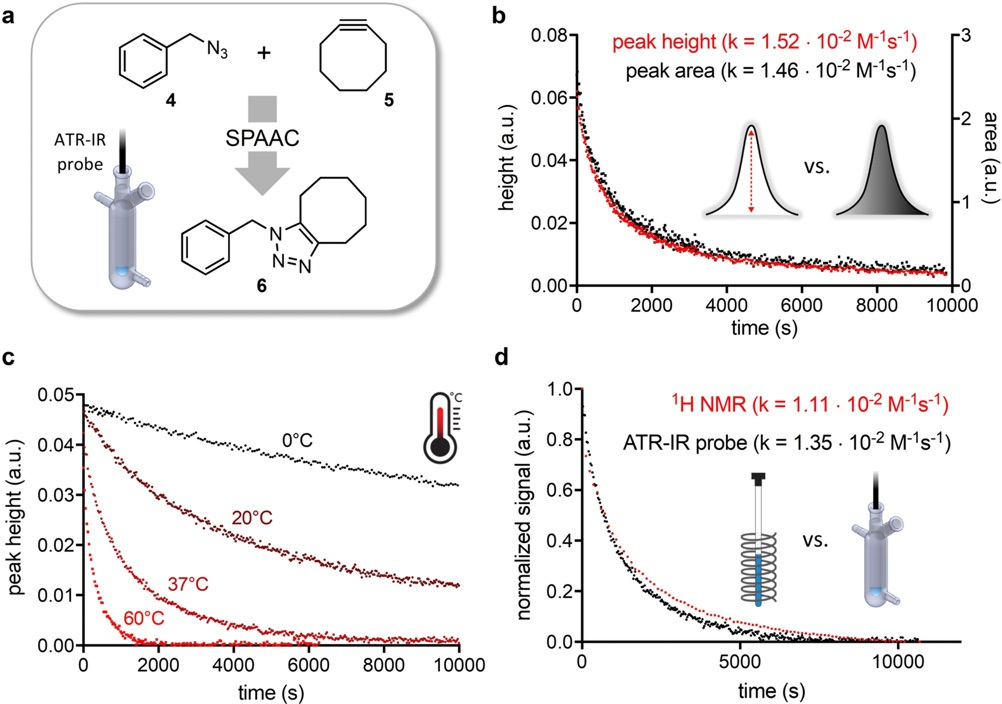
a) Investigation of the strain‐promoted azide alkyne cycloaddition (SPAAC) of **4** and **5** by inline ATR‐IR gave similar results for monitoring the reaction (37 °C, 100 mm in DMSO) based on peak height and area (b). c) Our setup enables measurements at different temperatures (100 mm, 0 °C to 37 °C in acetonitrile; 60 °C in DMSO) with results comparable to those obtained by NMR measurements (d) (in [D_3_]acetonitrile, 100 mm, 37 °C).

Measurements at different concentrations, ranging from 10 to 100 mm were conducted (see Supporting information). Although measurement at low mm concentrations (<25 mm) is possible, the signal‐to‐noise ratio is reaching the limit for accurate analysis. Therefore, a starting concentration of 50 mm or higher is recommended for reliable and reproducible measurements.

A big advantage of our setup is the ability to easily monitor reactions at different temperatures. We have been able to monitor the SPAAC ligation of **4** and **5** at different temperatures, ranging from 0 to 60 °C (Figure 3 c). Second order rate constants were determined to be in the range from 5×10^−4^ m ^−1^ s^−1^ at 0 °C to 5.83×10^−2^ m ^−1^ s^−1^ at 60 °C showing an overall increase of the reaction rate of approximately 150‐fold.

In addition, we have compared our results for monitoring by using inline ATR‐IR to commonly used ^1^H NMR measurements (Figure 3 d) by studying the reaction between **4** and **5** in acetonitrile at 37 °C. Both methods gave very similar results and comparable rate constants (1.11×10^−2^ m ^−1^ s^−1^ determined by NMR and 1.35×10^−2^ m ^−1^ s^−1^ determined by ATR‐IR). To evaluate the system's performance for the monitoring of faster ligations, measurements of the reaction between the highly reactive cyclooctyne bicyclo[6.1.0]non‐4‐yn‐9‐ylmethanol (BCN, **7**, *endo*‐isomer)^16^ and benzyl azide (**4**) were performed. The second order rate constant for this reaction in DMSO at 37 °C was determined to be 0.15 m ^−1^ s^−1^ (Figure 4).

**Figure 4:**
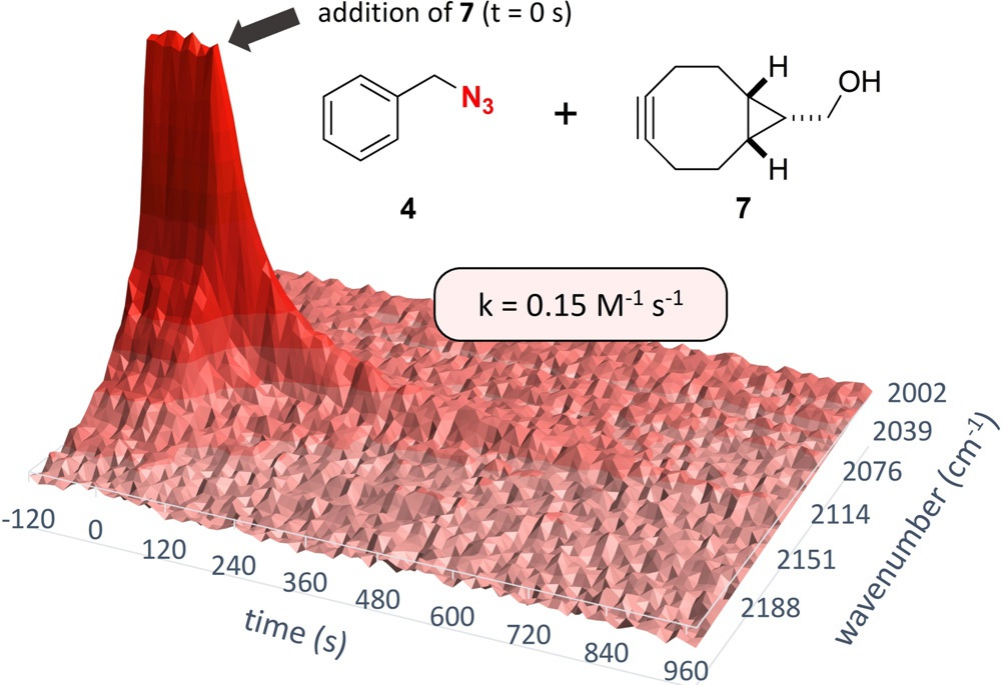
3D‐plot of the azide band during the ATR‐IR monitoring of the reaction of benzyl azide (**4**) and BCN (**7**) (DMSO, 100 mm, 37 °C).

To assess the applicability of our method for the monitoring of SPAAC in water and even complex biological fluids, IR spectra of water‐soluble 2‐azidoethanol (**8**) in both water (see Supporting Information) and human blood plasma (Figure 5 a) were measured at different concentrations. Both peak height and peak area of the azide band showed very good linear correlation for concentrations ranging from 20 to 200 mm in both solvents.

**Figure 5:**
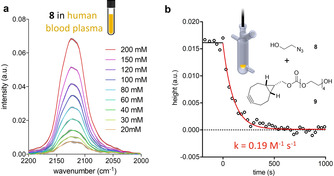
a) Azide signal in human blood plasma at 37 °C at different concentrations (gray areas indicate SD, *n*=3). b) Monitoring of the reaction between 2‐azidoethanol (**8**) and the PEGylated BCN derivative **9** in human blood plasma (20 °C, 100 mm).

Since the results did not reveal a significant difference between the performance in water and blood plasma, we proceeded to investigate the monitoring of SPAAC in human blood plasma. Therefore, the reaction of 2‐azidoethanol (**8**) and the water‐soluble PEGylated BCN derivative **9** at 100 mm concentration and 20 °C was followed (Figure 5 b). Despite a lower signal‐to‐noise ratio (in comparison to organic solvents), the decrease of azide signal could reliably be used for the determination of the rate constant (peak height: 0.19 m ^−1^ s^−1^ vs. peak area: 0.21 m ^−1^ s^−1^). Data for reaction monitoring at 50 mm and 37 °C (peak height: k=0.57 m ^−1^ s^−1^) is provided in the Supporting Information.

In summary, we have developed a method for inline ATR‐IR kinetic measurements of strain‐promoted azide alkyne cycloadditions enabling live reaction monitoring even in complex biological fluids such as blood plasma. The setup can be used for measurements at different temperatures and low reaction volumes. Data acquisition is possible during the addition of the reaction partner and therefore first data points are obtained right after the start of the reaction. A relatively short interval of 15 s between the scans enables the monitoring of fast SPAAC reactions (k>0.1 m ^−1^ s^−1^), whereas longer intervals can be used for slow conversions. Even though limited to azide concentrations >10 mm, this method can be used for kinetic investigation of fast bioorthogonal SPAAC ligations in complex reaction mixtures and environments, providing important information on the reactivity of the used reaction partners. Hence, we expect this method to find application in the fields of bioorthogonal chemistry and bioconjugation, and provide valuable insights regarding the kinetics of strain‐promoted azide alkyne cycloadditions.

## Conflict of interest

The authors declare no conflict of interest.

## Biographical Information

*Hannes Mikula obtained his PhD from TU Wien in 2014. Inspired by the emerging field of bioorthogonal chemistry, he then joined the lab of Ralph Weissleder at the Massachusetts General Hospital & Harvard Medical School as a postdoctoral fellow. In 2016 he returned to Vienna and since 2018 he is leading his own group (‘Molecular Chemistry & Chemical Biology’) at the Institute of Applied Synthetic Chemistry (TU Wien). His team focuses on the development of bioorthogonal tools and reactions for in vivo chemistry, diagnostics, and therapeutic strategies*.

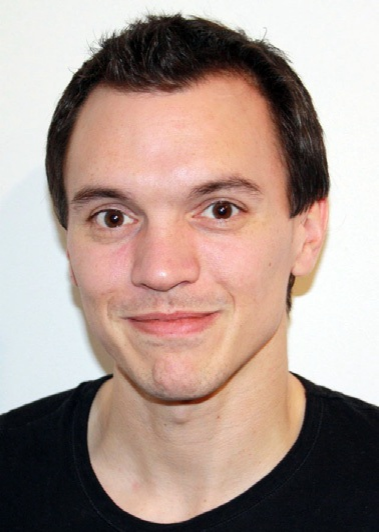

## Supporting information

As a service to our authors and readers, this journal provides supporting information supplied by the authors. Such materials are peer reviewed and may be re‐organized for online delivery, but are not copy‐edited or typeset. Technical support issues arising from supporting information (other than missing files) should be addressed to the authors.

Supplementary
