# Supplementary material for: Live Monitoring of Strain‐Promoted Azide Alkyne Cycloadditions in Complex Reaction Environments by Inline ATR‐IR Spectroscopy
Source: Chemistry. 2020 Mar 13;26(44):9851–4. doi: 10.1002/chem.201905478 (PMC7496163; doi:10.1002/chem.201905478)
Supplement: Supplementary file 1 — Supplementary [file CHEM-26-9851-s001.pdf]

Chemistry–A European Journal

Supporting Information

Live Monitoring of Strain-Promoted Azide Alkyne Cycloadditions in Complex Reaction Environments by Inline ATR-IR Spectroscopy

Dennis Svatunek,[a, b] Gottfried Eilenberger,[a] Christoph Denk,[a] Daniel Lumpi,[a] Christian Hametner,[a] Günter Allmaier,[b] and Hannes Mikula*[a]

Contents

Materials and Methods **S2**
Synthesis **S2**
BCN-PEG$_4$-OTrt (**11**) S2
BCN-PEG$_4$-OH (**9**) S3
Setup **S3**
ATR-IR measurements and kinetic experiments **S5**
General procedure S5
Data Analysis S5
Calibration curves in acetonitrile, water and human blood plasma S5
Measurements at different concentrations S7
Measurements at different temperatures S9
Comparison between NMR and ATR-IR S10
ATR-IR monitoring in human blood plasma S11
References **S12**

Materials and Methods

Unless otherwise noted, all reagents were purchased from commercial suppliers and used without further purification. Cyclooctyne (**5**),[1] BCN (**7**, endo-isomer),[2] 13,13,13-triphenyl-3,6,9,12-tetraoxatridecanol (**10**)[3] and 2-azidoethanol (**8**)[4] were prepared following known procedures. DCM was dried using PURESOLV-columns (Innovative Technology Inc.). All other solvents were distilled prior to use. Drying of organic solvents after extraction was performed using anhydrous Na_2SO_4 and subsequent filtration. Reactions were carried out under an atmosphere of argon in air-dried glassware with magnetic stirring. Sensitive liquids were transferred via syringe. Thin layer chromatography was performed using TLC alumina plates (Merck, silica gel 60, fluorescence indicator F254, or Merck, aluminium oxide neutral, fluorescence indicator F254). Preparative normal phase column chromatography was performed using a Büchi Sepacore Flash System (2 x Büchi Pump Module C-605, Büchi Pump Manager C-615, Büchi UV Photometer C-635, Büchi Fraction Collector C-660) using silica gel 60 (40-63 µm) as obtained from Merck and distilled or redistilled solvents.

^{1}H and ^{13}C NMR spectra were recorded on a Bruker AC 200 or a Bruker Avance UltraShield 400. Chemical shifts are reported in ppm (δ) relative to tetramethylsilane and calibrated using solvent residual peaks. Data is shown as follows: Chemical shift, multiplicity (s = singlet, d = doublet, t = triplet, q = quartet, quin = quintet, m = multiplet, br = broad signal), coupling constant (J, Hz) and integration. HRMS measurements were done on a Thermo Scientific MALDI LTQ Orbitrap mass spectrometer using α-cyano-4-hydroxycinnamic acid (CHCA) as matrix.

Synthesis

BCN-PEG$_4$-OTrt (11)

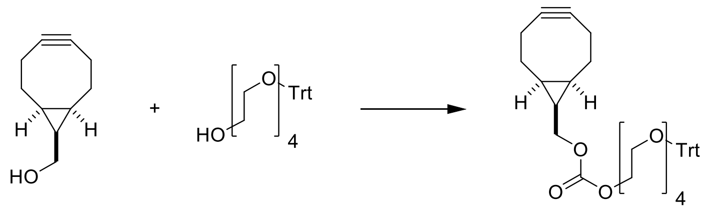

7 **10** **11**

To a stirred solution of triphosgene (0.19 g, 0.65 mmol) and pyridine (0.47 g, 6 mmol) in dry CH_2Cl_2 (2 mL) at -20 °C was dropwise added a solution of **10** (0.87 g, 2 mmol) in CH_2Cl_2 (2 mL). After stirring at -20 °C the mixture was allowed to reach RT. Subsequently BCN (**7**) (0.15 g, 1 mmol) was added. After stirring at RT overnight the reaction mixture was diluted with CH_2Cl_2 and washed twice with water. The organic phase was dried over Na_2SO_4 and concentrated *in vacuo*. The residual oil was purified by column chromatography (90 g silica gel, hexanes/ethyl acetate = 3:1) to afford 152 mg of **11** (25%) as a yellowish oil.

^{1}H NMR (200MHz, $CDCl_3$): δ 7.02-7.56 (m, 15 H), 4.04-4.25 (m, 4 H), 3.44-3.72 (m, 12 H), 3.16 (t, J = 5.2 Hz, 2 H), 1.97-2.37 (m, 5 H), 0.66-1.63 (m, 6 H); ^{13}C NMR (50 MHz, $CDCl_3$): δ 155.18 (s, 1 C), 144.02 (s, 3 C), 128.61 (d, 6 C), 127.66 (d, 6 C), 128.62 (d, 3 C), 98.67 (s, 1 C), 86.43 (s, 2 C), 70.70 (t, 1 C), 70.67 (t, 1 C), 70.59 (t, 3 C), 68.84 (t, 1 C), 66.81 (t, 1 C), 66.14 (t, 1 C), 63.23 (t, 1 C), 28.91 (t, 2 C), 21.28 (t, 2 C), 20.16 (d, 2 C), 17.31 (d, 1 C); HRMS: calcd. for $[M+Na]^+$ 635.2979, found 635.2990.

BCN-PEG$_4$-OH (9)

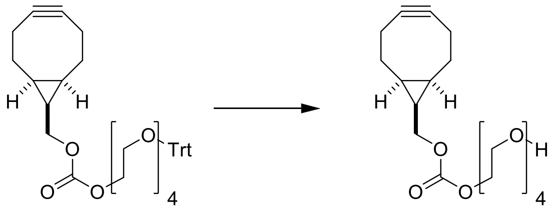

11 **9**

A solution of **11** (66 mg, 127 µmol) in 80% acetic acid (2 mL) was stirred at 40 °C for 2.5 h and subsequently cooled to 0 °C. The white precipitate was filtered of and washed with water. The combined filtrates were lyophilized to afford 34 mg of **9** (85%) as a colorless oil.

^{1}H NMR (200MHz, DMSO-d$_6$): δ 4.58 (br. s., 1 H), 4.11-4.25 (m, 4 H), 3.12-3.72 (m, 14 H), 1.93-2.36 (m, 6 H), 0.73-1.71 (m, 5 H); ^{13}C NMR (50 MHz, DMSO-d$_6$): δ 154.71 (s, 1 C), 98.96 (s, 2 C), 72.35 (t, 1 C), 69.82 (t, 1 C), 69.75 (t, 4 C), 68.19 (t, 1 C), 66.64 (t, 1 C), 65.66 (t, 1 C), 60.21 (t, 1 C), 28.50 (t, 2 C), 20,80 (t, 2 C), 19.73 (d, 2 C), 17.13 (d, 1 C); HRMS: calcd. for $[M+Na]^+$ 393.1884, found 393.1893.

Setup

For ATR-IR kinetic experiments a ReactIR 15 System from METTLER TOLEDO was used in combination with a DST Series AgX Fiber SiComp probe. As a reaction vessel a custom-made flask was used (Fig. S1, Fig. S2). A double mantle enables connection to a thermostat for accurate temperature control. An NS14 joint on top is used for inserting the ATR-IR probe and a second one for a temperature sensor and/or the addition of reagents.

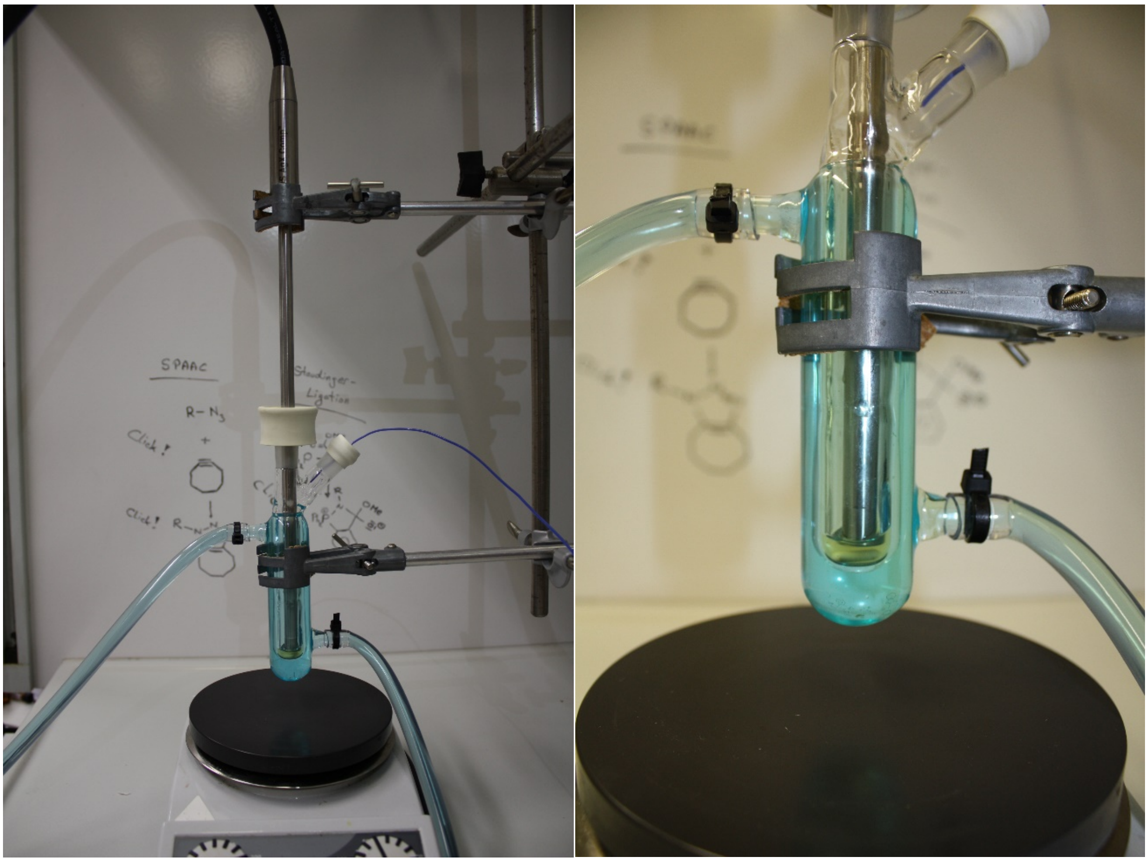

Figure S1. Double-walled IR reaction vessel with inserted ATR-IR probe.

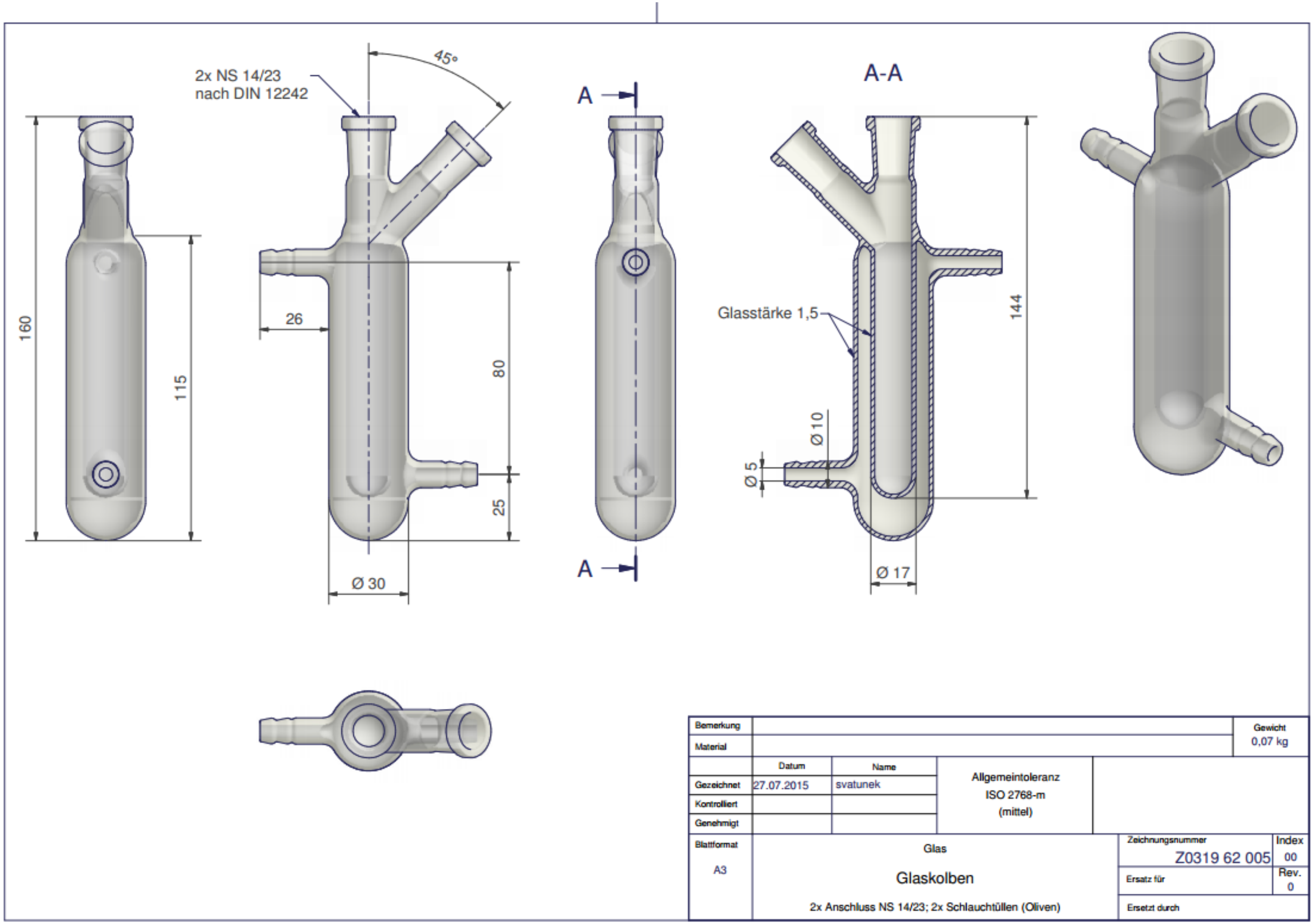

Figure S2. Construction drawings of the used reactor (values in mm).

ATR-IR measurements and kinetic experiments

General procedure

Baseline of pure solvent at the reaction temperature was recorded as background. The azide solution (0.9 mL) was stirred and the ATR-IR probe was immersed. After reaching a constant temperature the measurement was started. Then a solution of the 1,3-dipolarophile (0.1 mL) was added and this time point was set to t = 0 s for data analysis.

Data Analysis

Data was recorded and pre-processed using the iC IR Software 4.3 (Mettler Toledo). The interval between full scans was chosen based on the reaction rate, ranging from 15 s for fast reactions to 1 min for slower conversions. Baseline offset was performed using the value at 2000 cm^{-1}. Solvent background was subtracted and the signal region was drawn around the azide signal at ~2100 cm^{-1}. Both “Area to zero” and “Height to zero” time curves of that signal were exported. For determination of rate constants these curves were linearized by plotting 1/[azide] against time in seconds leading to a slope equal to second order rate constants of the reaction (Formula S1).

Formula S1: $$\frac{1}{[azide]} = \frac{1}{[azide_{t=0}]} + k * t$$

Calibration curves in acetonitrile, water and human blood plasma

Calibration was done for azides in acetonitrile, water and human blood plasma at 37 °C. Benzyl azide (**4**) was used for calibration in acetonitrile and 2-azidoethanol (**8**) for calibration in water and human blood plasma. Calibration was done for peak height and peak area. Data analysis was performed using PRISM 6 (Graphpad).

benzyl azide (4) in acetonitrile

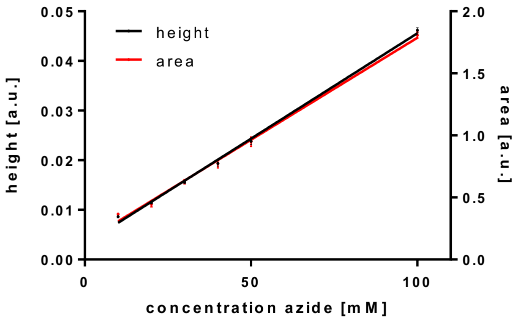

	peak height	peak area
Slope	0.0004243 ± 6.727e-006	0.01644 ± 0.0003216
Y-intercept when X=0	0.003141 ± 0.0003419	0.1429 ± 0.01635
X-intercept when Y=0	-7.402	-8.691
1/slope	2357	60.83
95% Confidence Intervals		
Slope	0.0004101 to 0.0004386	0.01576 to 0.01712
Y-intercept when X=0	0.002416 to 0.003866	0.1082 to 0.1775
X-intercept when Y=0	-9.377 to -5.538	-11.19 to -6.362
R^2	0.9960	0.9939

2-azidoethanol (8) in water

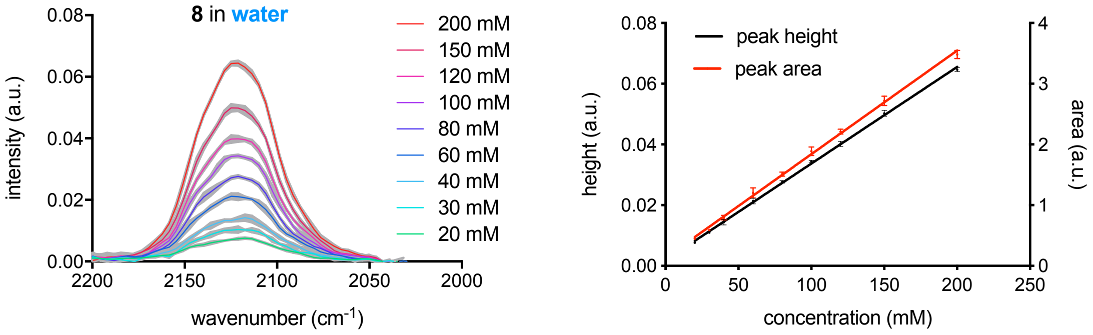

	peak height	peak area
Slope	0.0003185 ± 2.744e-006	0.01707 ± 0.0002119
Y-intercept when X=0	0.001842 ± 0.0002889	0.1320 ± 0.02231
X-intercept when Y=0	-5.785	-7.732
1/slope	3140	58.57
95% Confidence Intervals		
Slope	0.0003128 to 0.0003241	0.01664 to 0.01751
Y-intercept when X=0	0.001247 to 0.002438	0.08604 to 0.1780
X-intercept when Y=0	-7.772 to -3.858	-10.66 to -4.932
R^2	0.9981	0.9962

2-azidoethanol (8) in human blood plasma

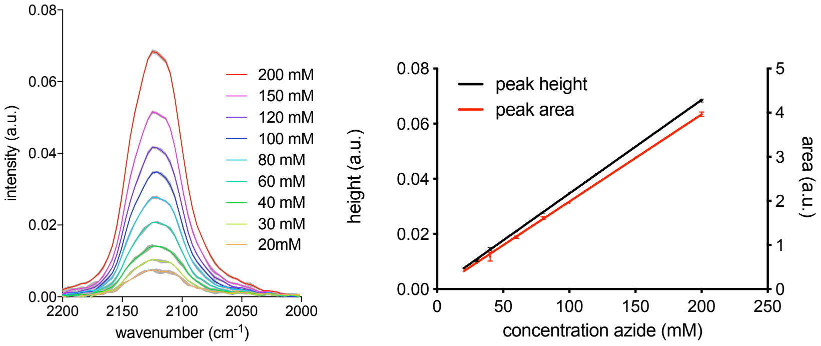

	peak height	peak area
Slope	0.0003397 ± 1.487e-006	0.01976 ± 0.0001702
Y-intercept when X=0	0.0006990 ± 0.0001566	0.008287 ± 0.01792
X-intercept when Y=0	-2.058	-0.4194
1/slope	2944	50.61
95% Confidence Intervals		
Slope	0.0003366 to 0.0003427	0.01941 to 0.02011
Y-intercept when X=0	0.0003765 to 0.001021	-0.02863 to 0.04520
X-intercept when Y=0	-3.030 to -1.100	-2.323 to 1.428
R^2	0.9995	0.9981

Measurements at different concentrations

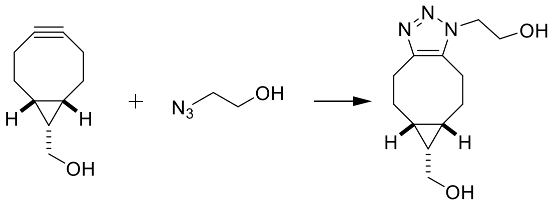

7 **8** **12**

The reaction of BCN (**7**) and 2-azidoethanol (**8**) in acetonitrile was monitored at different concentrations (10, 25, 50 and 100 mM) at 37 °C (Fig. S3, Table 1S).

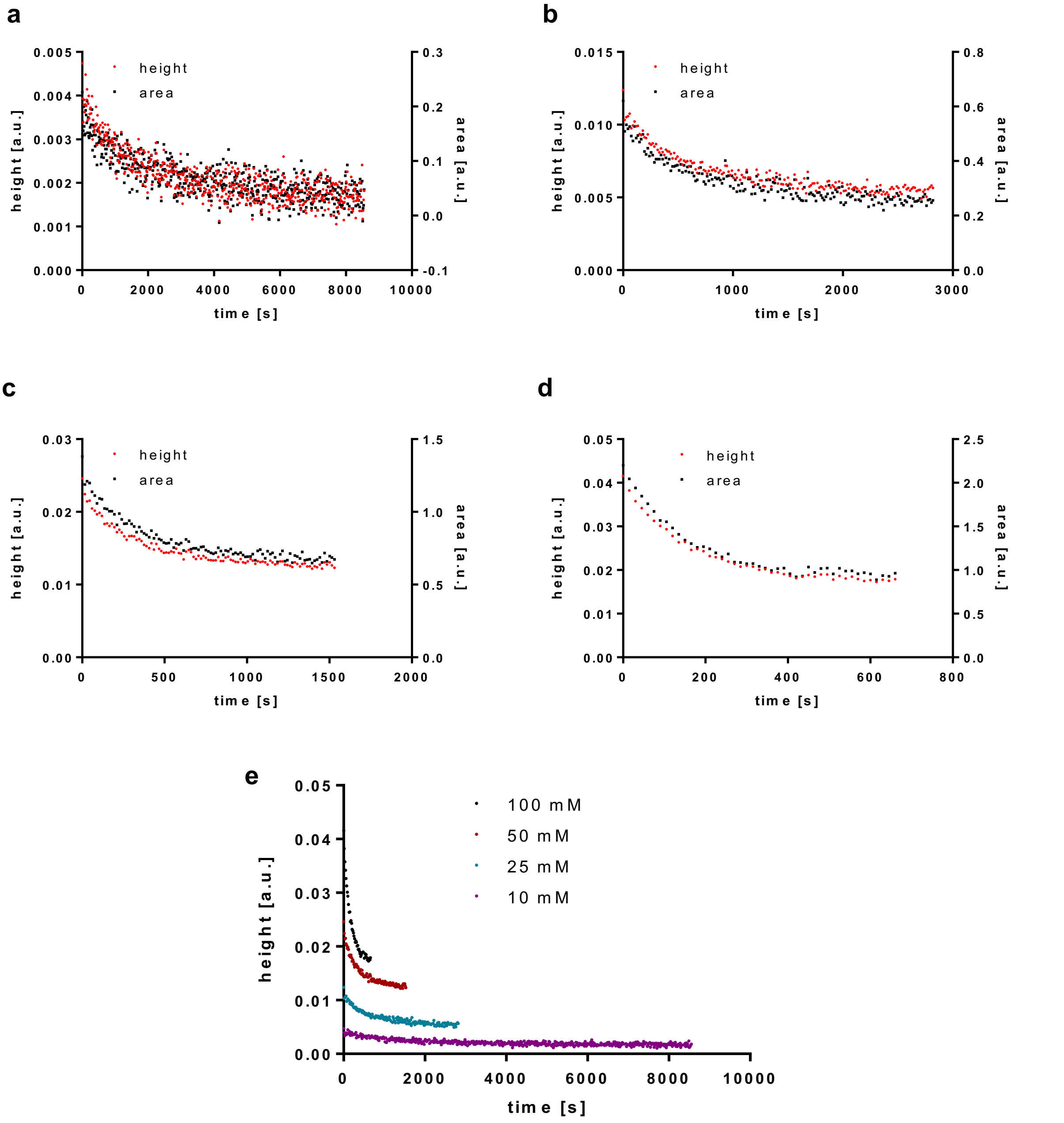

Figure S3. Reaction of **7** and **8** in acetonitrile at 37 °C with both reagents at **a)** 10 mM **b)** 25 mM **c)** 50 mM and **d)** 100 mM concentration; **e)** Comparison of measurements at different concentrations based on peak height.

Table S1. Determined rate constants for the reaction of **7** and **8** in acetonitrile at 37 °C.

Concentration (mM)	second order rate constant ($M^{-1}s^{-1}$)
**10**	4.61×10^{-2}
**25**	4.75×10^{-2}
**50**	5.03×10^{-2}
**100**	4.80×10^{-2}

Measurements at different temperatures

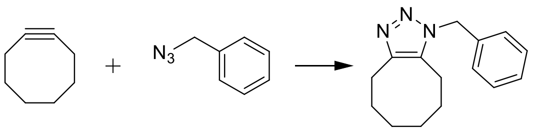

5 **4** **6**

The reaction of benzyl azide (**4**) and cyclooctyne (**5**) was monitored at different temperatures. 100 mM was chosen as starting concentration for both reagents Measurements were done at a concentration of 100 mM of both reagents in acetonitrile (0 °C, 20 °C and 37 °C) and DMSO (37 °C and 60 °C) (Fig. S4, Table S2).

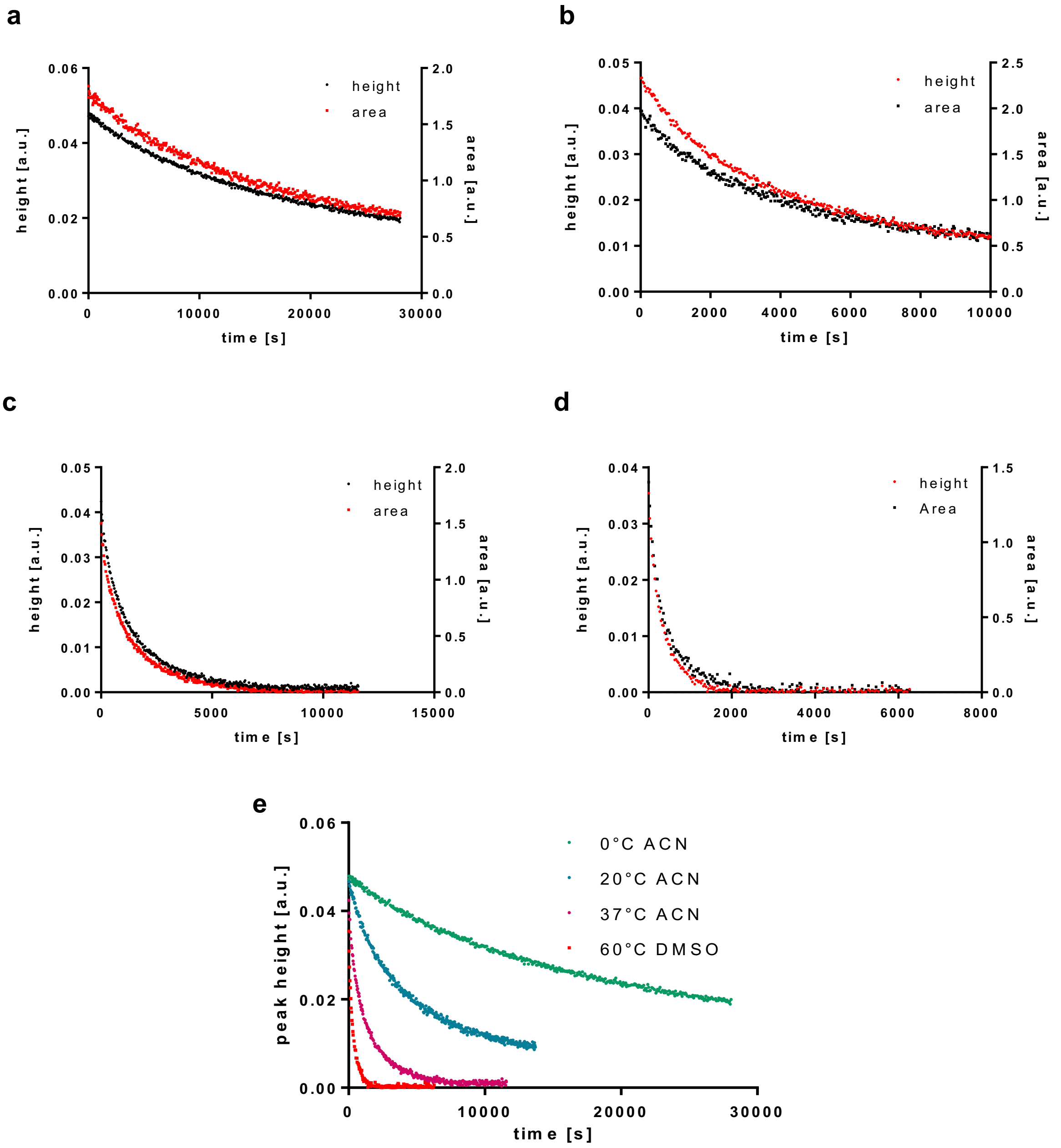

Figure S4. Reaction of **4** and **5** at 100 mM starting concentration at **a)** 0 °C in acetonitrile, **b)** 20 °C in acetonitrile, **c)** 37 °C in acetonitrile. and **d)** 60 °C in DMSO; **e)** Comparison of measurements at different temperatures based on peak height.

Table S2. Determined rate constants for the reaction of **4** and **5** at 100 mM.

Temperature (°C)	solvent	second order rate constant ($M^{-1}s^{-1}$)
**0**	acetonitrile	4.99×10^{-4}
**20**	acetonitrile	2.79×10^{-3}
**37**	acetonitrile	1.35×10^{-2}
**37**	DMSO	1.52×10^{-2}
**60**	DMSO	5.83×10^{-2}

Comparison between NMR and ATR-IR

For comparison with NMR kinetic experiments the reaction of cyclooctyne (**5**) and benzyl azide (**5**) was performed in acetonitrile and acetonitrile-d_3 for IR and NMR experiments, respectively.

NMR kinetic experiments were performed on a Bruker Avance UltraShield 400 at 37 °C. An NMR tube containing 0.6 mL of a 100 mM solution of benzyl azide (**4**) in dry acetonitrile-d_3 was put into the tempered NMR and shimmed. The tube was removed and one equivalent of cyclooctyne (**5**) was added. After vigorous shaking the tube was reinserted into the NMR and ^{1}H spectra were measured with an interval of 2 minutes. To determine the conversion of the reaction the singlets of the two hydrogens in benzylic position of benzyl azide (Ph-C$\underline{H_2}$-N_3) and the click product (Ph-C$\underline{H_2}$-triazole) were integrated. The chemical shifts of these protons are 4.42 ppm for benzyl azide and 5.48 ppm for the resulting triazole. ATR-IR kinetic experiments were performed as described above at a starting concentration of 100 mM and at 37 °C.

In case of ATR-IR measurements data points could be obtained during and right after addition of the cyclooctyne while in case of NMR measurements the first data point could only be obtained after 2 minutes (Fig. S5, Table S3).

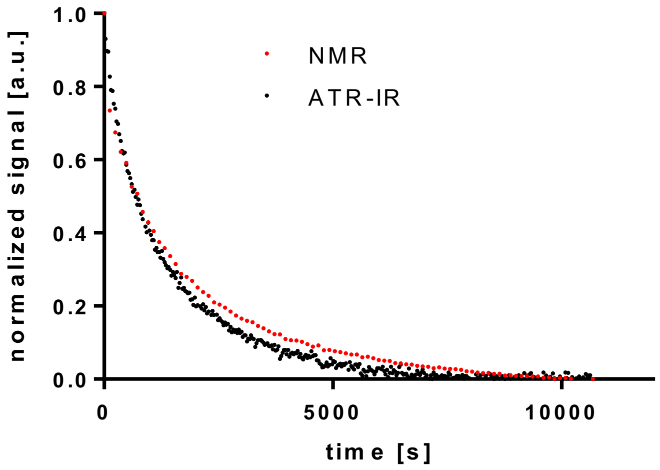

Figure S5. Comparison of ATR-IR (peak height) and NMR data.

Table S3. Determined rate constants for the reaction of **4** and **5** at 100 mM in acetonitrile by NMR and ATR-IR.

Modality	second order rate constant ($M^{-1}s^{-1}$)
**NMR**	1.11×10^{-2}
**ATR-IR**	1.35×10^{-2}

ATR-IR monitoring in human blood plasma

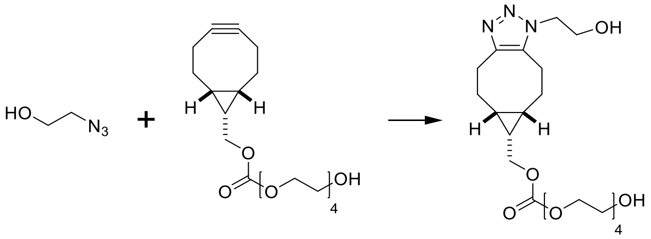

8 9 13

The reaction of the water-soluble BCN derivative **9** and water-soluble azide **8** was monitored in human blood plasma at 50 mM starting concentration at 37 °C and at 100 mM starting concentration at 20 °C (Fig. S6, Table S4).

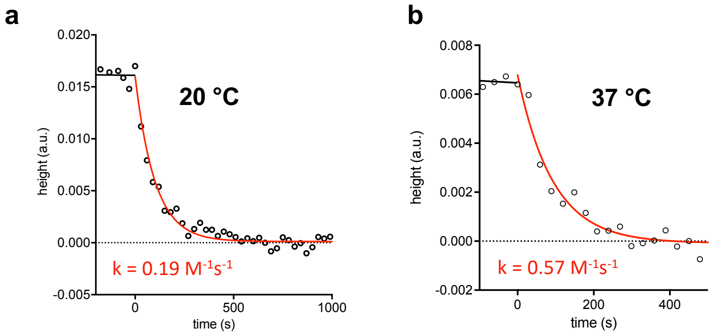

Figure S6. Monitoring of reaction between **8** and **9** in human blood plasma at a) 100 mM and 20 °C, and b) 50 mM and 37 °C (peak height).

Table S4. Determined rate constants for the reaction of **8** and **9** in human blood plasma.

Temperature (°C)	Concentration (mM)	second order rate constant ($M^{-1}s^{-1}$)
**37**	50	0.57
**20**	100	0.19

References

(1) Meier, H.; Voigt, E. Bildung und fragmentierung von cycloalkeno-1,2,3-selenadiazolen. *Tetrahedron* **1972**, 28, 187-198.

(2) Dommerholt, J.; Schmidt, S.; Temming, R.; Hendriks, L. J. A.; Rutjes, F. P. J. T.; van Hest, J. C. M.; Lefeber, D. J.; Friedl, P.; van Delft, F. L. Readily Accessible Bicyclononynes for Bioorthogonal Labeling and Three-Dimensional Imaging of Living Cells. *Angew. Chem. Int. Ed.* **2010**, 49, 9422-9425.

(3) Lumpi, D.; Braunshier, C.; Hametner, C.; Horkel, E.; Zachhuber, B.; Lendl, B.; Fröhlich, J. Convenient multigram synthesis of monodisperse oligo(ethylene glycols): effective reaction monitoring by infrared spectroscopy using an attenuated total reflection fibre optic probe. *Tetrahedron Lett.* **2009**, 50, 6469-6471.

(4) Pfaendler, H. R.; Weimar, V. Synthesis of Racemic Ethanolamine Plasmalogen. *Synthesis* **1996**, 1996, 1345-1349.
